# CSF flow measurement in the mesencephalic aqueduct using 2D cine phase-contrast MRI in dogs with communicating internal hydrocephalus, ventriculomegaly, and physiologic ventricular spaces

**DOI:** 10.3389/fvets.2024.1473778

**Published:** 2024-11-06

**Authors:** Daniela Farke, Francesca Dörn, Sebastian Schaub, Ella Wenz, Katharina Büttner, Martin J. Schmidt

**Affiliations:** ^1^Department of Veterinary Clinical Sciences, Small Animal Clinic – Neurosurgery, Neuroradiology and Clinical Neurology, Justus-Liebig-University, Giessen, Germany; ^2^Department of Veterinary Clinical Sciences, Small Animal Clinic – Surgery, Justus-Liebig-University, Giessen, Germany; ^3^Unit for Biomathematics and Data Processing, Faculty of Veterinary Medicine, Justus-Liebig-University, Giessen, Germany

**Keywords:** CSF flow dynamics, brachycephaly, canine, ventriculomegaly, internal hydrocephalus

## Abstract

**Background:**

Brachycephalic dogs are overrepresented with ventricular enlargement. Cerebrospinal fluid (CSF) flow dynamics are not completely understood. MRI techniques have been used for the visualization of CSF dynamics including phase-contrast imaging.

**Objectives:**

This study aimed to determine a causality between CSF flow and ventriculomegaly or hydrocephalus and to compare CSF flow dynamics among dogs with ventriculomegaly, internal hydrocephalus, and physiologic ventricles.

**Animals:**

A total of 51 client-owned dogs were included in the study.

**Methods:**

Magnetic resonance imaging (MRI)-based FLASH sequences and phase-contrast images of the brain were obtained, and the ROI was placed at the level of the mesencephalic aqueduct. ECG monitoring was performed parallel to MRI acquisition. Evaluation of flow diagrams and processing of phase-contrast images were performed using commercially available software (Argus VA80A, Siemens AG Healthcare Sector, Erlangen, Germany). Dogs were divided into three groups: Group 1 consisted of brachycephalic dogs with ventriculomegaly (group 1A) or internal hydrocephalus (group 1B), group 2 consisted of brachycephalic dogs with normal ventricles, and group 3 consisted of meso- to dolichocephalic dogs with normal ventricles.

**Results:**

Group 1 had a higher median V_rost_ (4.32 cm/s; CI: 2.94–6.33 cm/s) and V_caud_ (−6.1 cm/s, CI: 3.99–9.33 cm/s) than group 2 (V_rost_: 1.99 cm/s; CI 1.43–2.78 cm/s; V_caud:_ 2.91 cm/s, CI: 2.01–4.21 cm/s; *p* = 0.008; *p* = 0.03) and group 3 (V_rost_:1.85 cm/s, CI: 1.31–2.60 cm/s; V_caud_ − 2.46 cm/s, CI 1.68–3.58 cm/s; *p* = 0.01; *p* = 0.02). The median Vol_caud_ of group 1 (−0.23 mL/min, CI: 0.13–0.42 mL/min) was higher than that of group 2 (−0.09 mL/min, CI: 0.05 mL/min and 0.15 mL/min) (*p* = 0.03). Groups 1A and 1B did not differ in V_caud_, V_rost_, Vol_caud_, and Vol_rost_. Group 1A and 1B showed a higher median V_rost_ (4.01 cm/s, CI: 2.30–7.05 cm/s; 5.94 cm/s, CI: 2.16–7.88 cm/s) than group 2 (1.85 cm/s, CI: 1.24–2.80 cm/s.) (*p* = 0.03; *p* = 0.004).

**Conclusion and clinical importance:**

Increased CSF flow velocities in rostral and caudal directions are present in dogs with ventriculomegaly and internal hydrocephalus compared to normal controls.

## Introduction

Brachycephalic dogs and cats are frequently observed with internal hydrocephalus showing clinical signs and enlarged ventricles. They are also more likely to have ventriculomegaly, which is the enlargement of the ventricles (1, 2). Despite the absence of neurological signs, canine ventriculomegaly is associated with a loss of white matter and reduced periventricular perfusion, which implies an active distension of the lateral cerebral ventricles with a negative effect on the periventricular white matter (3, 4). Breeding for brachycephalic skull features has resulted in a fundamental change in the neurocranium of dogs and cats (5–9). A premature fusion of skull sutures and synchondroses of the skull base in brachycephalic breeds was found in different dog and cat breeds that leads to a shortening of the skull base, a deformation of the skull vault, and a reduction of the endocranial capacity (5, 8, 10, 11). Higher grades of brachycephaly were correlated with more severe ventricular dilation and internal hydrocephalus in dogs and cats (6, 12). Based on these findings, a pathophysiologic relation between a brachycephalic skull morphology and the development of ventricular distension must be assumed, but a definitive cause–effect relation was not shown, until now.

Phase-contrast MRI is a non-invasive technique for measuring CSF flow dynamics (13, 14) and can be used for quantitative CSF flow measurement in the mesencephalic aqueduct in both humans and dogs (15–18). In this study, the technique was used to investigate potential differences in CSF flow in the mesencephalic aqueduct in brachycephalic and mesocephalic dogs with normal ventricular dimensions compared to brachycephalic dogs with ventriculomegaly or hydrocephalus.

## Materials and methods

### Study population

The study population consisted of dogs with internal hydrocephalus that were referred for diagnosis and surgical intervention. Flow measurement sequences were performed as part of the diagnostic workup. Clinically normal dogs with brachycephalic and mesocephalic skull conformation were recruited for the study. Only dogs with internal hydrocephalus (group 1B) showed neurological clinical signs localized to the forebrain; all dogs of the other groups were normal on neurological examination. These dogs were also examined in the scope of another research study (19–21), which did not interfere with our research aims. Dogs with structural intracranial lesions other than enlargement of the ventricular system and animals with aqueductal stenosis were excluded from the study. Animals that showed a heart frequency below 60 and above 120 beats per minute during the MRI examination were therefore also excluded from the study, as previously described (18, 22). The study population was divided into three groups:

Group 1: Brachycephalic dogs with internal hydrocephalus or ventriculomegaly.
Group 1A:(ventriculomegaly): Dogs normal on neurological examination and with enlarged ventricles.
Group 1B:(internal hydrocephalus): Dogs with clinical signs localized to the forebrain and with enlarged ventricles.
Group 2:Brachycephalic dogs with normal ventricular dimensions (23).
Group 3:Meso- to dolichocephalic dogs with normal ventricular dimensions (23).

### Ethical clearance

This study was conducted strictly according to the recommendations in the Guidelines for Care and Use of Laboratory Animals of the German Animal Protection Law. Clinically normal dogs with and without ventriculomegaly were scanned with the approval of the Committee on the Ethics of Animal Experiments of the Justus Liebig University Giessen and the Regierungspraesidium Hessen (Permit number: 22-2684-04-02-075/14). The study was conducted with the owners’ written informed consent.

### Magnetic resonance imaging

CSF flow measurement using non-invasive phase-contrast MRI was performed at the Clinic for Small Animals of the Justus Liebig University in Giessen from July 2017 to December 2020. MRI acquisition was performed by a board-certified senior radiologist (SS). A standard anesthetic protocol was used for MRI examination and surgical procedure in each animal. Diazepam (0.5 mg/kg) was administered intravenously into a venous catheter (20 gauge), which was placed in the right or left cephalic vein. Anesthesia was induced with propofol (2–4 mg/kg, IV). Dogs were endotracheally intubated, and anesthesia was maintained with 2% isoflurane in oxygen. Dogs were placed in sternal recumbency during the MRI procedure.

Imaging was performed using a 3.0 Tesla Magnetom Verio (Siemens, Germany) and a four-channel flexible surface coil or a 15-channel knee coil depending on the size of the dog. The images included at least transversal T1 (TR 588, TE 15, slice thickness 1 mm) and transverse, sagittal, and dorsal T2-weighted images (turbo spin echo, TR 2900 ms, TE 120 ms, slice thickness 3 mm) to exclude central nervous system (CNS) disease or morphological abnormalities of the brain.

**Figure 1:**
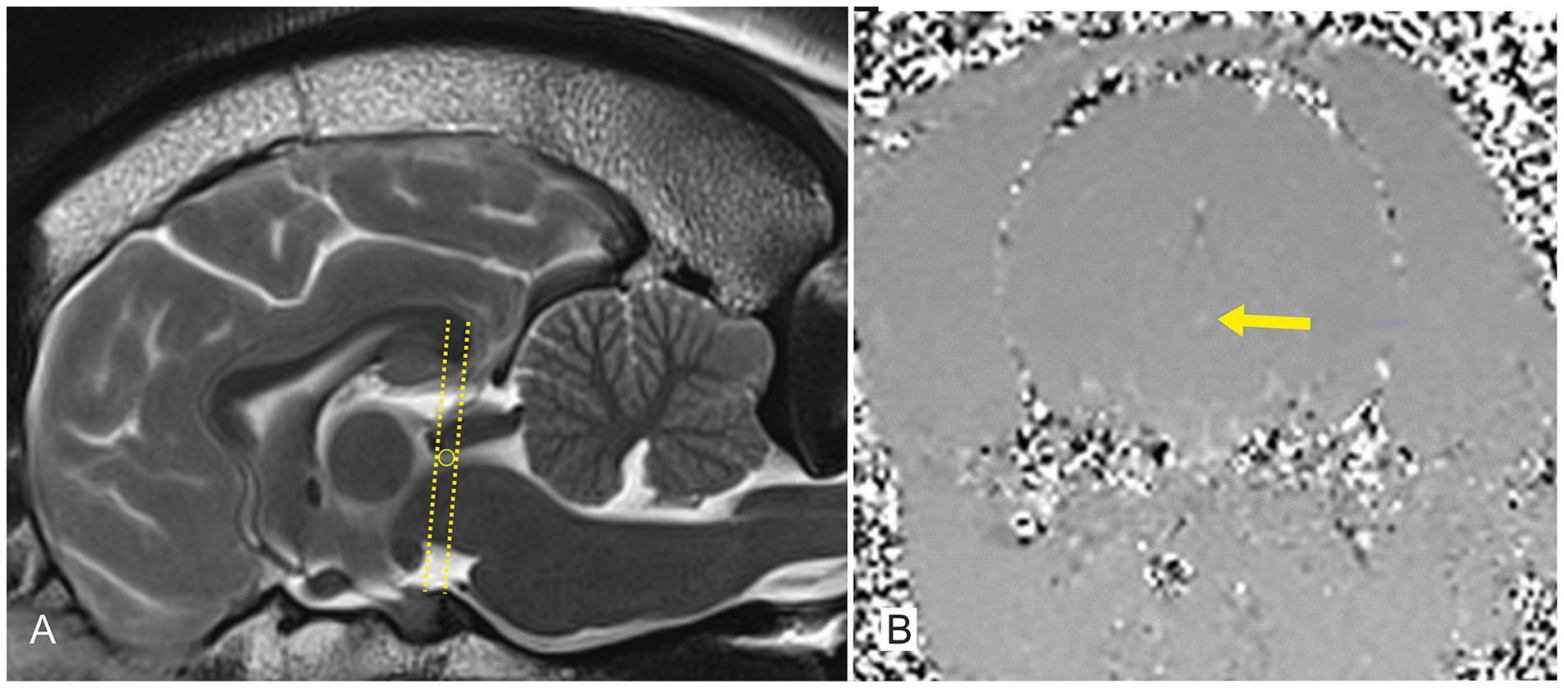
A T2-weighted sagittal image (A) and a phase-contrast image in the transversal plane (B) of a 2-year-old French Bulldog from group 2. The T2-weighted MRI (A) demonstrates the level of the mesencephalic aqueduct (lines and circle) at which further phase-contrast images were obtained in the transverse plane (B). The region of interest is pointed out within the phase-contrast image (arrow).

FLASH sequences were performed in a transverse plane perpendicular to the mesencephalic aqueduct, and phase-contrast images were generated. The region of interest (ROI) was placed at the level of the mid-mesencephalic aqueduct (Figure 1). FLASH sequence imaging parameters were obtained as follows: FoV: 180 mm; TR: 30.36 ms, TE: 10.92 ms, flip angle: 10°, slice thickness: 6 mm, phase-contrast: 100%, voxel: 0.7 × 0.7 × 6 mm, basis contrast: 256, venc: −3 – 3, matrix 256 × 256. Efforts were spent to keep surrounding brain tissue out of the ROI. In order to exclude systematic errors by imperfect suppression of eddy currents due to brain motion, another measurement in the mesencephalic tegmentum was obtained (24) representing the apparent velocity in a stationary tissue of no flow and subtracted from the apparent velocities in the ROI (25). Parallel to MRI acquisition, the cardiac cycle was derived and recorded using MR-compatible electrodes (Kendall ECG electrodes, Cardinal Health™; Norderstedt, Germany). The FLASH sequence was started with a solid electrocardiographic signal with a constant heart rate of >60 and < 120 beats per minute (18, 22). Measurements were determined from several heart cycles. After the measurement, the transverse images of the FLASH sequence and the 2D phase contrast were obtained.

### Image analysis

The evaluation of the flow diagrams and the processing of the phase-contrast images and data collection were carried out using the “Argus VA80A” (Copyright 2004 Siemens). Image analysis was performed using a single investigator (FD), who was instructed and supervised by a board-certified radiologist. Directional programming was defined as negative for flow from rostral to caudal (caudal direction) and positive for flow from caudal to rostral (rostral direction) through the aqueduct. The software generated a time velocity curve and measured maximum velocity in the caudal direction (V_caud_), maximum velocity in the rostral direction (V_rost_) in cm/s, and the maximal CSF net flow volume in the caudal direction (Vol_caud_) and rostral direction Vol_rost_ in ml/min.

### Statistical analysis

Statistical analysis was performed using a commercial statistical software package (Base SAS® 9.4 Procedures Guide: Statistical Procedures, 2nd edition ed. Statistical Analysis System Institute Inc., Cary, NC, United States). The covariables age, bodyweight, and heart rate were obtained for each dog. Variables consisted of V_caud_, Vol_caud_, V_rost_, and Vol_rost_ and were also assessed for each dog. V_caud_, Vol_caud_, V_rost_, Vol_rost_, and the covariables age and bodyweight were not normally distributed. In order to obtain a normal distribution, a log_10_ transformation was performed on these data. An analysis of covariance was performed to determine the influence of each group and the covariates on the variables. Once the analysis of covariance had been validated, it was performed for all four variables. The first analysis of covariance was performed between groups 1, 2, and 3. Another analysis of covariance was then performed to compare group 1A, group 1B, and group 2.

The *p*-values of the pairwise comparisons were processed using Bonferroni correction to adjust the significance level for all variables. For all statistical tests, a significance level of 0.05 was applied. A difference between the groups was assumed with a *p*-value of <0.1 and a highly significant difference at *p* < 0.01.

## Results

### Animals

A total of 84 flow measurements were performed, and 33 dogs were excluded from further analysis due to a heart rate of <60 or > 120 beats per minute. A total of 51 dogs were included in the study. Phase-contrast MRI enabled the assessment of CSF flow in all these dogs.

Group 1: Internal hydrocephalus or ventriculomegaly with brachycephalic skull conformation (14); 1A: ventriculomegaly (*n* = 8); 1B: internal hydrocephalus (*n* = 7). The mean age was 24 months (5–120 months), the mean body weight was 7.7 kg (2–30 kg), and the mean heart rate was 98.5 beats per minute (63–114 beats per minute) in this group.
Group 2: Physiologic ventricular spaces with brachycephalic skull conformation (*n* = 16). The mean age was 48 months (12–180 months), the mean body weight was 9.6 kg (3–37 kg), and the mean heart rate was 97.1 beats per minute (70–120 beats per minute) in this group.
Group 3: Physiologic ventricular spaces with meso- or dolichocephalic skull conformation (*n* = 20). The mean age was 72 months (12–144 months), the mean body weight was 20.2 kg (7–50 kg), and the mean heart rate was 95.9 beats per minute (62–119 beats per minute) in this group.

**Figure 2:**
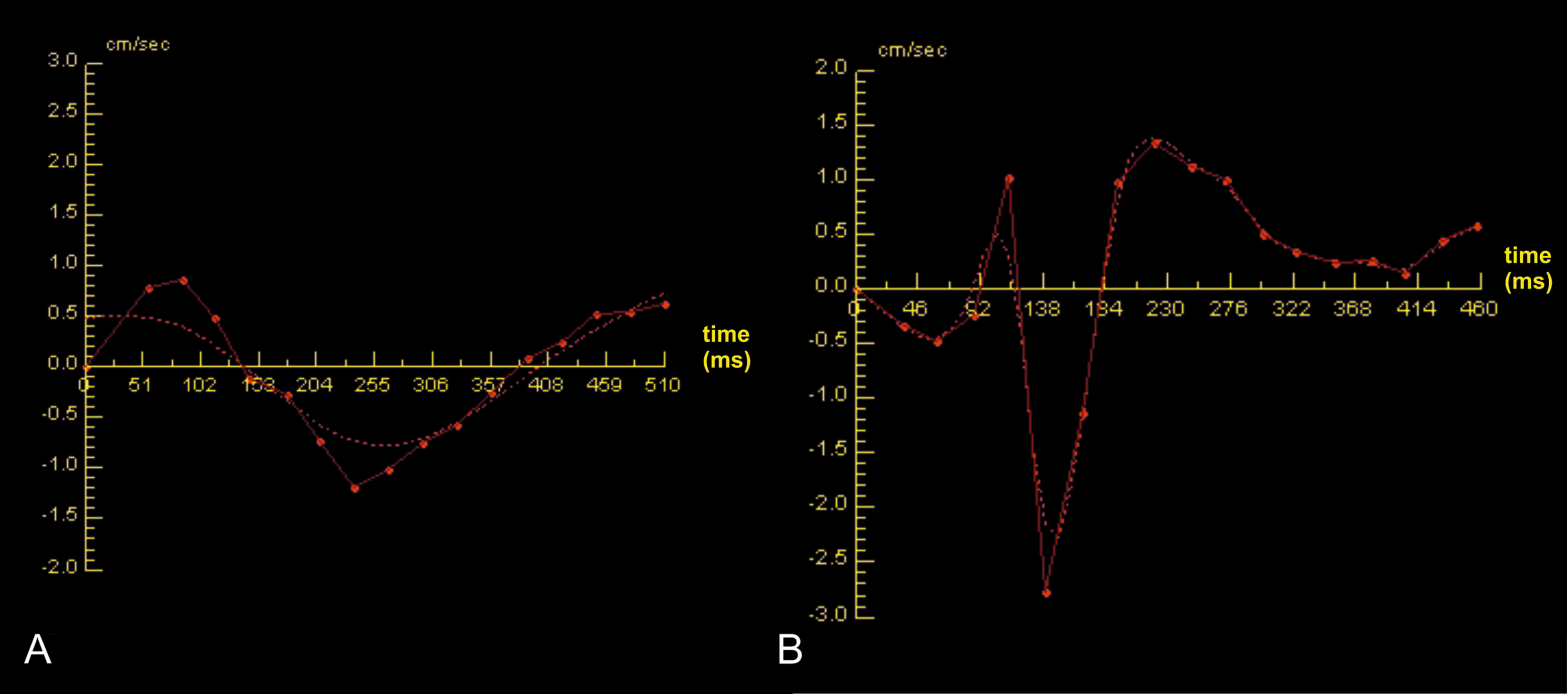
A biphasic sinusoidal flow curve over one cardiac cycle is shown in a Chihuahua of group 2 (A) and a Chihuahua of group 1B (B). The red dots represent the individual data sets per phase-contrast image. The x-axis corresponds to the time in milliseconds over one cardiac cycle and the y-axis to the velocity in centimeters per second. Positive values above the zero line correspond to diastolic values and represent the rostral flow direction. In contrast, negative values below the zero line correspond to the systolic values, with a caudal direction of flow. The dashed line represents the spline for curve modeling. Higher flow velocities can be observed in group 1B (B) compared to group 2 (A).

A typical sinusoidal pattern of CSF flow was observed during the cardiac cycle (Figure 2). The respective flow measurements of each group are given in Table 1.

**Table 1:** Dog breeds including their age, body weight, and measured flow parameters in group 1A (brachycephalic dogs with internal hydrocephalus), group 1B (brachycephalic dogs with ventriculomegaly), group 2 (brachycephalic dogs with normal ventricular dimensions), and group 3 (mesocephalic dogs with normal ventricular dimensions).

Group	Breed	Age (months)	Body weight (kg)	V_rost_ (cm/s)	V_caud_ (cm/s)	Vol_caud_ (ml/min)	Vol_rost_ (ml/min)
1A	Chihuahua	24	3	4.7	−9.1	−0.234	0.476
1A	Chihuahua	120	2	3.8	−4.4	−0.067	0.131
1A	English Bulldog	84	30	2.8	−4	−0.286	0.051
1A	Shih Tzu	10	5	1.7	−2.2	−0.097	0.074
1A	Chihuahua	24	2	7.6	−11.2	−0.515	0.382
1A	English Bulldog	24	28	3.8	−2.8	−0.048	0.043
1A	Rusky toy	48	2	2.5	−2.2	−0.057	0.003
1A	French Bulldog	60	9	9.8	−2.6	−1.994	0.499
1B	Chihuahua	24	4	3.1	−9.7	−0.539	0.043
1B	French bulldog	12	14	25.4	−18.8	−0.307	0.239
1B	Chihuahua	12	2	13.3	−27.5	−0.571	0.309
1B	Maltester	36	3	1.4	−1.8	−0.065	0.084
1B	Chihuahua	36	3	2.1	−4.1	−0.204	0.07
1B	French Bulldog	5	7	2	−2.3	−0.134	0.03
1B	Chihuahua	60	2	2.6	−2.8	−0.112	0.103
2	Cavalier King Charles spaniel	36	6	4.7	−8.7	−0.257	0.164
2	Boxer	48	30	2	−2	−0.012	0.056
2	Cavalier King Charles spaniel	84	9	2.6	−3.8	−0.151	0.091
2	Old English bulldog	108	37	2.1	−4	−0.228	0.063
2	French bulldog	72	16	3.2	−5.3	−0.191	0.151
2	Chihuahua	12	3	1.5	−2.1	−0.051	0.089
2	Yorkshire terrier	60	3	1.8	−2.1	−0.146	0.066
2	Pekingese	36	8	1.2	−2.3	−0.036	0.02
2	Chihuahua	48	6	2.5	−3.4	−0.056	0.067
2	Shih Tzu	12	3	0.9	−1.8	−0.098	0.038
2	Chihuahua	60	3	0.6	−0.8	−0.005	0.018
2	Chihuahua	24	3	8.6	−1.2	−0.26	0.304
2	Pug dog	36	9	1.4	−1.4	−0.052	0.022
2	French bulldog	48	10	1.3	−2.7	−0.136	0.003
2	Maltese	24	5	2	−2.2	−0.124	0.157
2	Yorkshire terrier	180	3	1.3	−2.1	−0.164	0.038
3	Rhodesian Ridgeback	60	50	2.2	−4.8	−0.3	0.071
3	German Shepherd dog	72	39	2.1	−2.2	−0.135	0.013
3	Fox terrier	96	12	2.5	−2.2	−0.061	0.052
3	Labrador retriever	72	41	3.4	−3.5	−0.054	0.231
3	Beagle	48	11	1.9	−3.4	−0.078	0.153
3	Labrador retriever	36	33	2.4	−4.3	−0.22	0.112
3	Dachshund	72	7	2.8	−3.3	−0.165	0.058
3	Mixed breed dog	36	34	2.3	−3.4	−0.166	0.068
3	Miniature schnauzer	96	7	1	−1.8	−0.079	0.012
3	Dachshund	36	9	5.5	−6.7	−0.066	0.259
3	Jack Russel terrier	96	9	1.6	−1.8	−0.194	0.009
3	Labrador retriever	132	23	2	−2.6	−0.109	0.035
3	Bull terrier	12	12	1.2	−1	−0.015	0.037
3	Tibet terrier	120	15	1.7	−3.3	−0.315	0.002
3	Rhodesian Ridgeback	72	36	5.2	−5	−0.232	0.097
3	Dachshund	60	10	1.2	−1.8	−0.161	0.013
3	Mixed breed dog	24	14	3.6	−4.3	−0.17	0.124
3	Small Münsterländer	84	24	2.1	−2.7	−0.036	0.013
3	Mixed breed dog	72	8	0.9	−1	−0.023	0.009
3	Mixed breed dog	144	10	0.9	−1.8	−0.1	0.02

Group 1 showed a significantly higher median V_rost_ than group 2 (group 1: 4.32 cm/s; CI: 2.94–6.33 cm/s; group 2: 1.99 cm/s; CI 1.43–2.78 cm/s; *p* = 0.03). There was also a significant difference between groups 1 and 3, with group 1 showing a significantly higher median V_rost_ than group 3 (1.85 cm/s, CI: 1.31–2.60 cm/s; *p* = 0.02). There was no significant difference between the median V_rost_ of groups 2 and 3 (*p* > 0.05).

Group 1 showed a significantly higher median V_caud_ than group 2 (group 1: −6.1 cm/s, CI: 3.99–9.33 cm/s; group 2: −1.99 cm/s, CI 1.43–2.78 cm/s; *p* = 0.008). Group 1 also showed a significantly higher median V_caud_ than group 3 (−2.46 cm/s, CI 1.68–3.58 cm/s) (*p* = 0.01). There was no significant difference between the median V_caud_ of groups 2 and 3 (*p* > 0.05).

The median Vol_rost_ of group 1 (0.07 mL/min), group 2 (0.05 mL/min), and group 3 (0.05 mL/min) did not show any significant differences among the groups (*p* = 1). The median Vol_caud_ of group 1 (−0.23 mL/min, CI: 0.13–0.42 mL/min) was significantly higher than the median Vol_caud_ of group 2 (−0.09 mL/min, CI: 0.05 mL/min and 0.15 mL/min; *p* = 0.03). A comparison of group 1 and group 3 with a median Vol_caud_ of −0.09 mL/min revealed a tendency of difference among these two groups, but it was not significant (*p* = 0.08). There was no significant difference between group 2 and group 3 (*p* > 0.05).

Groups 1A and 1B showed a higher median V_rost_ (group 1A: 4.01 cm/s, CI: 2.30–7.05 cm/s; group 1B: 5.94 cm/s, CI: 2.16–7.88 cm/s) than group 2 (1.85 cm/s, CI: 1.24–2.80 cm/s; *p* = 0.03; *p* = 0.004).

Group 1A had a median V_rost_ of 4.01 cm/s (CI: 2.30–7.05 cm/s) that was not significantly different from group 1B with a median V_rost_ of 5.94 cm/s (CI: 2.16–7.88 cm/s; *p* = 0.83). Group 1A showed a significantly higher median V_rost_ than group 2 (1.85 cm/s; CI: 1.24–2.80 cm/s; *p* = 0.03). There was also a significant difference between the median V_rost_ of groups 1B and 2 (*p* = 0.004). Group 1A had a median V_caud_ of −5.27 cm/s, which was not significantly different from group 1B with a median V_caud_ of −6.3 cm/s (*p* = 1). There was no significant difference between group 1A and the median V_caud_ of group 2 (−2.73 cm/s; *p* = 0.29). A comparison between the median V_caud_ of group 1B and group 2 also did not show any significant difference (*p* = 0.2).

The median Vol_rost_ of group 1A (0.09 mL/min), group 1B (0.08 mL/min), and group 2 (0.05 mL/min) did not show any significant differences among the groups (*p* = 1). The median Vol_caud_ of group 1A was −0.18 mL/s and showed no significant difference compared to group 1B with a Vol_caud_ of −0.24 mL/min (*p* = 1).

## Discussion

A brachycephalic skull morphology has a profound influence on many organ systems in dogs including the brain (26, 27) and its ventricular system. Ventriculomegaly is often observed in brachycephalic dogs (3, 28), which raises the question of whether there is a causal relationship between brachycephalic skull features and the development of ventricular enlargement. Using phase-contrast MRI, we evaluated CSF flow velocities in the mesencephalic aqueduct of different groups of dogs and found an increased flow velocity in brachycephalic dogs with ventriculomegaly and internal hydrocephalus compared to both brachycephalic and mesocephalic dogs with physiologic ventricular dimensions.

As mentioned in the introduction, the skull conformation of brachycephalic animals is associated with premature closure of one or more skull sutures, which is referred to as craniosynostosis (8, 10, 11). Children with brachycephalic skull morphologies based on craniosynostoses often have abnormal morphology of the cranial base, causing jugular foramen stenosis and subsequent impairment of venous drainage. This, in turn, results in reduced reabsorption of CSF into the venous system, increased intraventricular pressure, and ventricular distension of variable degrees (29–32). Surgical widening of the jugular foramen was proven to lead to a reduction in internal hydrocephalus, which proves the pathophysiologic association (33). Decreased volume of the jugular foramen potentially causing venous congestion was also described in brachycephalic dog breeds (34) and was suggested to be associated with an enlargement of the cerebral ventricles (34–36).

Another pathogenic factor in children with craniosynostosis is a reduced cranial capacity (37, 38), which has important implications for the arterial “*windkessel*” function in the basal cisterns and for general brain compliance. Arterial distension in the basal cisterns and brain expansion during systole transform a pulsatile blood flow in the arterial ring (circle of Willis) and basilar artery to a smooth linear blood flow to the cerebral arterioles and capillaries (39). The systolic filling of the brain with blood causes brain parenchyma expansion, which, in turn, reduces the cerebral ventricle volume. This is suggested to be the driving force of CSF through the ventricular system (40, 41). Impaired space for the cerebral blood vessels and the parenchyma to expand can result in augmented pulsatile blood flow through the parenchyma and thereby a hyperdynamic CSF flow through the ventricles that cannot be fully drained through the mesencephalic aqueduct (42–46). A higher flow velocity in the aqueduct may offer a limited compensatory mechanism, but increased pumping of CSF out of the lateral and third ventricles likely results in partial reflection of the CSF pulse wave from the aqueduct back toward the third and lateral cerebral ventricles. Such an alteration of CSF flow dynamics was shown to be associated with the development of various forms of hydrocephalus in humans (15, 47–49). Again, a reduced cranial capacity was also found in dogs and cats with brachycephaly and craniosynostoses, and the same mechanisms described for humans with this skull growth disease were proposed to cause ventricular distension and hydrocephalus in animals (5, 6, 10, 50). The findings in the present study may further support this theory.

CSF flow through the aqueduct is bidirectional. Tachy- and bradycardia can result in changes in CSF flow dynamics, with tachycardia leading to reduced flow and bradycardia leading to increased flow within the mesencephalic aqueduct (22, 51). Severe tachycardia can result in the termination of measurements; therefore upper and lower reference values are chosen to account for a better comparison and reduce the risk of false measurements (18, 22). During the cardiac systole, there is a flow from the third ventricle toward the fourth ventricle, while the reverse occurs during diastole. In the dogs with internal hydrocephalus and ventriculomegaly, the caudally directed net flow volume (Vol_caud_) and caudal flow velocity (V_caud_) were higher than those in groups 2 and 3. This is consistent with the findings in humans with communicating internal hydrocephalus; however, published data in humans are quite inconsistent. While some authors did find increased caudal flow volume (52, 53), others documented increased rostral flow volume (54) or found both increased caudal flow volume and rostral flow volume in their cohorts (55). The measured data in group 1 would indicate that not enough CSF volume can exit the ventricular system through the lateral apertures, and a higher volume than usual flows back to the third ventricle through diastole. This CSF contributes to the overload of the third and lateral cerebral ventricles and to their progressive distension.

It is interesting to note that differences in flow velocity and volume between brachycephalic dogs with normal ventricular dimensions (group 2) and meso−/dolichocephalic dogs with normal ventricular dimensions (group 3) were not identified. Following the hypothesis that aberrant CSF flow is a pathogenetic factor in the development of ventricular distension and brachycephaly is promoting the hyperdynamic flow, it could be expected to measure a subsequent increase of flow parameters between groups, with normal brachycephalic dogs having higher CSF flow velocity and volume than normal mesocephalic dogs and brachycephalic dogs with ventriculomegaly having a higher velocity than brachycephalic dogs with normal ventricular dimensions. Finally, it could be expected to find a higher flow in brachycephalic dogs with internal hydrocephalus compared to brachycephalic dogs with ventriculomegaly. The lack of difference between normal brachycephalic dogs and mesocephalic dogs might be due to higher grades of brachycephaly in group 1 than in group 2. Evaluation of a significant difference in cranial indices between the groups would have been ideal, but the groups included many different dog breeds with different grades of brachycephaly in general. A comparison of homogenous groups of the same dog breed would have been ideal, but it was not possible during this study. Furthermore, the measurement in MRI only provides a snapshot, taken under general anesthesia and in a lying position, which potentially has an influence on measured parameters and does not reflect the conditions in the awake and standing animal. However, an alternative explanation for the lack of difference between normal brachycephalic dogs and mesocephalic dogs may be that the hypothesis concerning reduced cranial compliance and venous congestion in brachycephalic dogs in general only explains a part of the pathogenetic factors that cause ventricular distension.

The lack of a difference between groups 1A and 1B was also striking. It was suggested that ventriculomegaly might be a compensated or arrested form of hydrocephalus (3, 37). Based on the finding that both conditions have an abnormally high CSF flow, it would be possible that ventriculomegaly might be progressive, and affected dogs could be at risk of developing further ventricular distension in the future and should therefore be monitored. Further studies are necessary to investigate if a cutoff value can be found that helps to identify dogs that are at risk for further ventricular expansion and to justify repeated MRIs in these dogs.

Phase-contrast MRI is a comparably new technique in veterinary medicine. Two previous pilot studies used the technique in laboratory beagles and provided normative measurements as a reference (18, 56). It is interesting to note that peak velocities in normal mesocephalic dogs measured in the present study were higher (median 1.85 cm/s) than those in mesocephalic dogs measured in the other two studies (0.92; ± 0.5 cm/s (18) and 0.76 ± 0.43 cm/s (56)). However, there is an overlap of the measurements of all studies. CSF flow values can also vary in humans (57). First, the peak flow velocity can differ according to the level of the mesencephalic aqueduct at which the measurement was made. The aqueduct is funnel-shaped with the narrowest lumen at the rostral entry zone, while the caudal exit zone has the widest lumen. Based on our assessment, the measurements in the two studies mentioned and our own were taken at the same area of the mesencephalic aqueduct, making significant differences based on this unlikely. As in humans, variations in CSF flow measurements may be attributed to the difference in body size (58), which can influence head and brain size. Our control group included dogs with a mean body weight of 20 kg. This was much higher than in standard laboratory beagles. Age could also play a role, as a study comparing CSF flow velocity in human infants found a higher CSF flow velocity than in adults (59). Additionally, a linear increase in peak velocity with age has been observed in adult humans (60).

## Conclusion

Brachycephalic dogs with both internal hydrocephalus and ventriculomegaly have increased caudally directed flow velocity and volume compared to brachycephalic and mesocephalic dogs with normal ventricular dimensions. As brachycephalic dogs with ventriculomegaly have the same increased flow as dogs with internal hydrocephalus, repetitive MRI might be indicated to control further ventricular distension.

## Data Availability

The raw data supporting the conclusions of this article will be made available by the authors, without undue reservation.
